# Prolonged physical isolation, agonistic behaviour, and human resilience in pandemic times

**DOI:** 10.3389/fpubh.2025.1542344

**Published:** 2025-03-18

**Authors:** Giuseppina Marsico, Claudio Russo

**Affiliations:** ^1^Department of Human, Philosophical and Educational Sciences, University of Salerno, Fisciano, Italy; ^2^Federal University of Bahia, Salvador, Brazil; ^3^Stress Management and Therapy Clinic, Naples, Italy

**Keywords:** physical isolation, pandemic bias, agonistic behaviour, health, resilience

## Abstract

With the purpose of enhancing a comprehensive approach to healthcare, public health initiatives have moved from managing the pandemic response towards an increased understanding of the sequelae, including but not limited to mental health issues triggered by societal limitations and precautionary measures. The long-term effects of the COVID-19 pandemic lie in the health system’s capacity to promote a renewed sense of healthy communities, strengthen individual resilience, and mitigate environmental stressors in the future. Under these terms, the pandemic breakdown has been discussed in relation to the public health crisis and physical isolation resulting from SARS-CoV-2 disease.

## Introduction

Social stressors are well-known to interfere with individual thoughts, triggering negative emotions and affecting human behaviour (1). Traditionally, the relationship between aggressive behaviour and social deprivation showed a response variability in laboratory studies of non-human animals and humans (2). Specifically, different types and models of aggression were proposed in the study of neural circuits behind the expression of aggressive behaviour, including environmental influences and the occurrence of social cues, emotions (e.g., fear or anxiety), motivational systems, and pleasure (3).

Since the fight against the SARS-CoV-2 pandemic has led us to 2 years of liminal feelings for the unknown consequences and cycle of the disease, human survival response to the infection transmission has resulted in a long-term impact on mental health (4). Nevertheless, expressed emotional states, one’s lived experience, the healthcare system crisis, transnational policy interventions, and individual responses have exacerbated pre-existing health inequity and increased social disparities, which may affect human resilience (5).

## Agonistic behaviour through the lens of contemporary science

In psychological terms, human agonistic behaviour may occur when external and/or internal stimuli elicit emotional processes, cognitive interpretation of events, or fight-or-flight responses. Previous studies in animal ecology have found long-lasting evidence that agonistic behaviour reduces reproduction and fertility and, conversely, it increases mortality and facilitates social dispersion (6). However, fear may drive aggressive behaviour, either in terms of primary or combined emotions expressed by human beings (7).

To develop a new framework on human agonistic behaviour, reinforced research efforts should move forward with the traditional model of cause-and-effect relationship (8), whereby a study’s focus should consider the health policy implications, the private and public dimensions, and the spatial distribution across dispersed geographical areas. Hence, the global effects of the pandemic are likely to represent “collective entities,” namely distinct groups of individuals who are culturally responsive and whose actions are individually based on the “disease,” the “policy,” and the “economy” of the pandemic.

According to the proposed definition, multi-omics or social entities are meant to exist separately from other things. It follows that collective entities should be addressed as something having an independent existence, namely all the integrative entities, either individual or collective, which belong to the process of agonistic behaviours. Hence, our understanding of entities should be more focused on processes rather than their own existence.

Unveiling the complex ontology of individual biology on human society would identify discrete entities in past or future pandemics, including those collective entities that are either cohabitating or disengaging. In terms of pandemic outcomes, agonistic behaviour is hereby introduced at either the individual or population level.

## Introducing the SARS-CoV-2 model of socio-behaviour analysis

**Figure 1:**
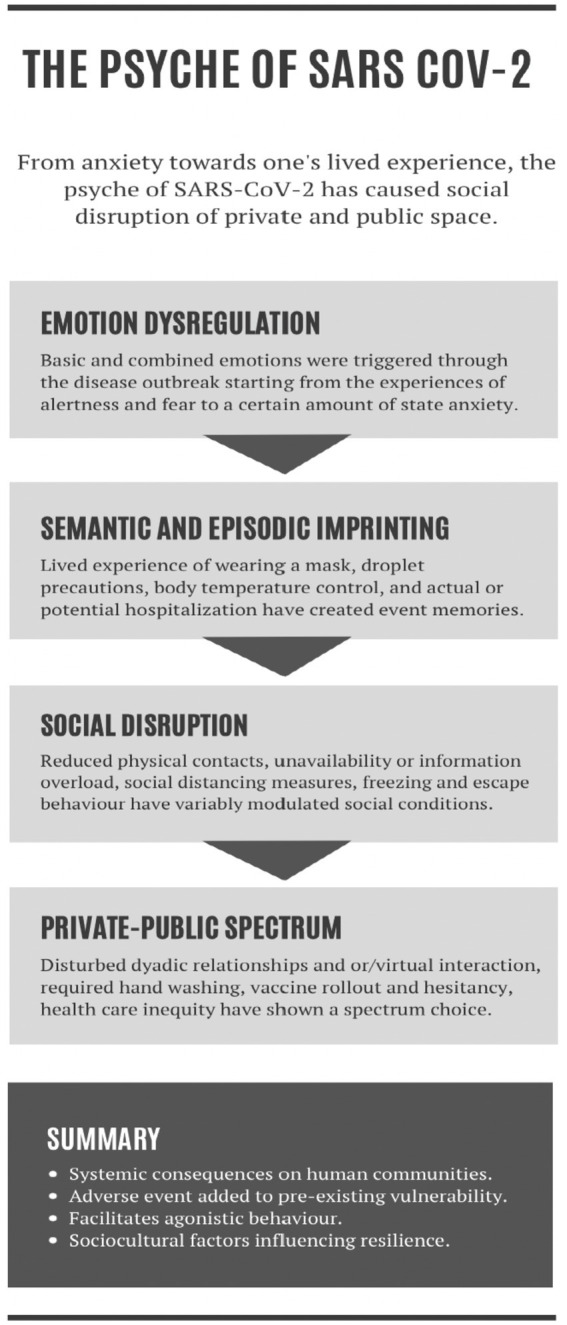
Psyche of SARS-CoV-2.

First, the pandemic has resulted in emotional dysregulation, evidenced by manifestations of fear, anxiety, irritability, and frustration. These emotional responses are automatic and well-established reactions to deprivation. Second, experiencing the pandemic has likely resulted in episodic and semantic imprinting. This phenomenon involves space-oriented and one-time exposure to event memories, which are conveyed through a more general understanding of one’s lived experiences. Third, self-isolation, quarantine, and limited physical interactions have exacerbated social deprivation through the enforcement of lockdown policies and social distance measures. Fourth, the immediate and delayed effects of the pandemic have led to unprecedented consequences on both the private and public spectrums. All four nodes of this model have produced a pattern of recovery (the “policy”), either in terms of individuals healing from the illness (the “disease”) or societies recovering from economic constraints (the “economy”). A four-node representation of what we call the psyche of SARS-CoV-2 is shown in Figure 1.

## Understanding the social isolation of human ecosystems

The ongoing changes in how we interpret the environment have involved the replacement of natural spaces in response to ever-evolving human needs and new modalities of adaptation. Technological and economic infrastructures have added more complexity to cyclical patterns whilst combining previous health risks from exposure to new environments (9).

Following the SARS-CoV-2 pandemic, our research priorities should include the prolonged period of physical isolation and consider what the unforeseeable outcomes could have been in terms of agonistic behaviours. The intermittence of physical isolation may feasibly have a negative impact over prolonged periods or during human developmental time. Furthermore, the prolonged uncertainty over the disease and its transmission appeared to reinforce the effects of physical isolation on an individual basis (“disease”) whilst influencing the real-time experiences amongst those collective entities (“economy” and “policy”).

Nevertheless, unwarranted generalisations may raise controversies and debates about whether the scientific evidence gathered on an individual basis (micro level of analysis) is used to explain societal events of collective entities (macro level of analysis). In particular, the latter consequences refer to the physical isolation exacerbated by the “disease” and the “policy” on communities, whilst agonistic behaviour relies on the social dispersal of the “economy” resulting from the SARS-CoV-2 pandemic. Indeed, research efforts to unveil the relationship between social isolation and perceived loneliness have shown the presence of complex proteomic networks, associations with morbidity and mortality profiles, and heterogeneity in health outcomes (10).

## Discussion

By considering the reiteration of the events throughout human history that have inspired either societal change or defeat, we argue it is time for immediate action on public health policy. We have paid particular attention to one of the possible health outcomes of the COVID-19 pandemic, namely aggressive or defensive behaviour at individual, community-based, or global levels. In the post-pandemic world, we might suggest revising a dominant view when promoting individual and community health against the unified global threats (e.g., climate change, SARS-CoV-2, and war) and the divided global market or competing commercial interests involved.

However, unveiling individual and global phenomena in this era requires psychological science to provide its own traditional methods and novel strategies. Three levels of analysis were presented to argue how the “disease,” the “policy,” and the “economy” of the pandemic have shaped what we call the psyche of SARS-CoV-2. Our aim for proposing a new analysis model was to reflect upon the aggressive view of human behaviour and to interpret the complex societal patterns of human resilience (11).

Along with individual’s readiness for positive change, the pandemic has triggered emotional dysregulation, created episodic and semantic imprinting, and generated social disruption over the private and public spectrum. Those collective entities, which also constitute the more socially disadvantaged ecosystems compared to the others, might jeopardise their own agonistic behaviour and be less likely to show collective resilience over time. As a result, agonistic behaviour might unintentionally increase systemic biases in medical research and policy.

Beyond the factors affecting an individual’s resilience, we question what impact the pandemic has on global health systems and the social significance of human-induced actions, including the expression of agonistic behaviours worldwide.

In 1986, the Seville Statement on Violence concluded that the biological foundations of individual aggressive behaviour do not cause the war itself, whilst a historical attempt was made to prevent the confusion and misuse of either individual attitudes or political warfare (12).

By referring to collective entities as multi-omics or social entities, are the pandemic sequelae related to agonistic behaviour or showing an increase in the number of human casualties? For this purpose, new research is recommended as a crucial step to address a falsifiable and scientific integration of health, education, and culture (13).

## Data Availability

The original contributions presented in the study are included in the article/supplementary material, further inquiries can be directed to the corresponding author.
